# Reference bias in the Illumina Isaac aligner

**DOI:** 10.1093/bioinformatics/btaa514

**Published:** 2020-05-21

**Authors:** Alex J Cornish, Daniel Chubb, Anna Frangou, Phuc H Hoang, Martin Kaiser, David C Wedge, Richard S Houlston

**Affiliations:** Division of Genetics and Epidemiology, The Institute of Cancer Research, London, SM2 5NG 2, UK; Division of Genetics and Epidemiology, The Institute of Cancer Research, London, SM2 5NG 2, UK; Nuffield Department of Medicine, Big Data Institute University of Oxford, OX3 7LF 3, UK; Molecular Diagnostics Theme, Oxford NIHR Biomedical Research Centre, Oxford, OX3 7LF 3, UK; Division of Genetics and Epidemiology, The Institute of Cancer Research, London, SM2 5NG 2, UK; Division of Molecular Pathology, The Institute of Cancer Research, London SM2 5NG 5, UK; Nuffield Department of Medicine, Big Data Institute University of Oxford, OX3 7LF 3, UK; Molecular Diagnostics Theme, Oxford NIHR Biomedical Research Centre, Oxford, OX3 7LF 3, UK; Manchester Cancer Research Centre, University of Manchester, M20 4GJ, UK; Division of Genetics and Epidemiology, The Institute of Cancer Research, London, SM2 5NG 2, UK

To the Editor,

The Isaac pipeline described in the *Bioinformatics* article ‘Isaac: ultra-fast whole-genome secondary analysis on Illumina sequencing platform’ (Raczy *et al.*, 2013) has been used in cancer sequencing studies (Burns *et al.*, 2018; Quigley *et al.*, 2018) and in the ongoing UK 100 000 Genomes Project (100KGP) (Turnbull *et al.*, 2018). Whilst Isaac has been benchmarked with respect to variant calling (Raczy *et al.*, 2013), there has been less extensive evaluation of its suitability for other analyses routine to cancer genomics.

Estimating the fraction of cancer cells with individual somatic mutations is central to cancer genome studies, including characterization of clonal architecture (Dentro *et al.*, 2017). Estimation of these cancer cell fractions (CCFs) is however contingent on unbiased assessment of the fraction of reads supporting variant allele frequencies (VAFs). We demonstrate that VAFs computed by Isaac are biased by the preferential soft clipping of reads supporting non-reference alleles, with deleterious consequences on downstream analyses reliant on unbiased CCF estimation.

Reads supporting heterozygous single-nucleotide polymorphism (SNP) reference and alternate alleles can be expected to occur with equal probability when sequencing normal tissue. Due to limited sequencing depth, the exact number of reads supporting reference and alternate alleles will not be equal in many instances, even when the aligner is unbiased. It can be expected however that the median VAF of a large number of heterozygous SNPs will be 0.5. We assessed heterozygous SNP VAF distributions in whole genome sequencing (WGS) data from the germline of 25 multiple myeloma (MM) tumor-normal pairs aligned to GRCh38Decoy assembly using Isaac v03.16.02.19. Germline variants were called using Starling v2.4.7 (Raczy *et al.*, 2013) and VAFs were calculated directly from alignment files using alleleCount (Van Loo *et al.*, 2010). Median VAFs per sample ranged from 0.478 to 0.479 (Fig. 1A), with this consistent skew indicating that Isaac can exhibit bias toward the reference allele.

**Fig. 1.**
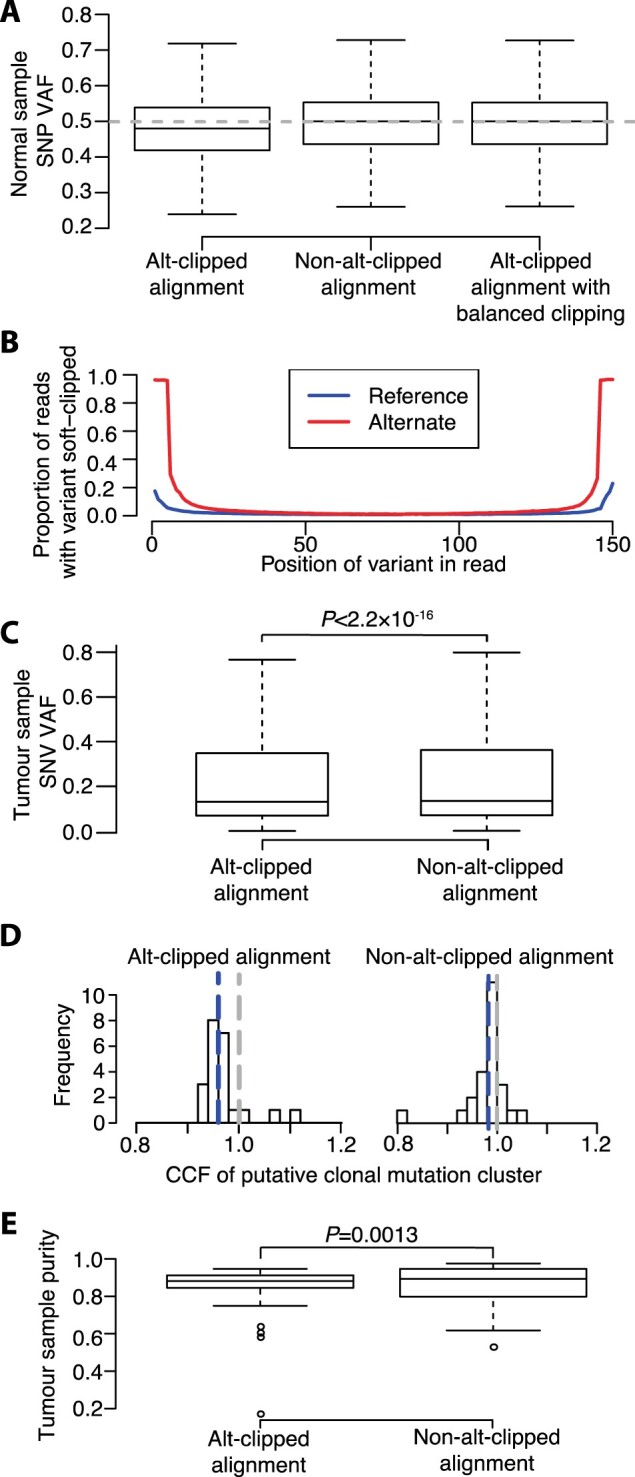
Evidence of reference bias from Isaac. (**A**) Heterozygous SNP VAF distributions in sequencing data from 25 normal samples. Dashed line represents expected median VAF of 0.5. Whiskers extend 1.5 times inter-quartile range and values outside of this range are not shown. (**B**) Proportion of reads covering SNP positions supporting the reference (blue line) and alternate (red line) alleles with that read position soft clipped. (**C**) SNV VAFs from 25 tumor-normal pairs. (**D**) CCFs of clonal mutation clusters identified by DPClust. Blue and grey dashed lines denote the median putative clonal mutation cluster CCF and a CCF of 1, respectively. (**E**) Ccube sample purity estimates. Distribution differences assessed using Wilcoxon Signed-Rank test

Isaac has a parameter (–clip-semi-aligned) that invokes the soft clipping of reads at each end until a stretch of five consecutive bases are matched with the reference sequence (here, we term this ‘alt-clipping’ to distinguish from soft clipping performed for other reasons). This parameter is present in all Isaac versions after v01.13.06.20 and was used to align the 25 normal samples. To test whether alt-clipping is responsible for the reference bias exhibited by Isaac, we re-aligned the 25 normal samples with Isaac without alt-clipping. When alt-clipping was not performed, the median VAF of heterozygous SNPs in each sample equaled 0.500 (Fig. 1A), thereby showing that alt-clipping introduces reference bias.

Alt-clipping results in the clipping of the majority of reads supporting the alternate allele where the variant position is within five bases of either read end (Fig. 1B). Fewer reads supporting the reference allele are soft clipped and VAFs therefore become biased towards the reference allele. If the preferential soft clipping of reads supporting the alternate allele is responsible for the reference bias, then we would expect the effect to be negated if the ends of all reads were soft-clipped by five bases. To further validate the effect of alt-clipping, we therefore soft-clipped five bases at each end of all reads in the alt-clipped alignments (here, we term this ‘balanced-clipping’). The median VAF of heterozygous SNPs in balanced-clipped alignments equaled 0.500 in each sample (Fig. 1A), demonstrating that the preferential clipping of reads supporting the alternate allele introduced by alt-clipping causes reference bias.

To assess the effect of alt-clipping on the analysis of cancer genomes, we aligned the tumor MM WGS data using Isaac with and without alt-clipping. Somatic single nucleotide variants (SNVs) were called using Strelka v2.4.7 (Kim *et al.*, 2018). Unlike SNPs in normal samples, we do not know the true VAF of SNVs in tumor samples, as they can be affected by copy number aberration, normal sample contamination and clonal heterogeneity. SNV VAFs from alt-clipped alignments were however lower than SNV VAFs from the same samples from alignments generated without alt-clipping (*P* < 2.2 × 10^−16^; Fig. 1C), indicating that alt-clipping also affects somatic SNV VAFs.

To test the effect of alt-clipping on subclonal reconstruction, we ran Battenberg and DPClust, which uses a Dirichlet process to model subclonal fractions (Nik-Zainal *et al.*, 2012). If SNV VAFs do not exhibit allelic bias, we would expect DPClust to identify clusters of mutations with CCFs centered on 1, representing clonal mutations. When DPClust was run using alignments generated with alt-clipping, the median CCF of putative clonal mutation clusters (defined as the cluster with a CCF closest to 1) was 0.959, compared to 0.983 when run using alignments generated without alt-clipping (Fig. 1D).

Finally, we assessed the effect of alt-clipping-induced reference bias on tumor sample purity estimation. Sample purities estimated using Ccube (Yuan *et al.*, 2018) were smaller when computed using alt-clipped alignments than non-alt-clipped alignments (*P* = 1.3 × 10^−3^; Fig. 1E), demonstrating that alt-clipping also affects purity estimation.

Reference bias introduced by Isaac through alt-clipping can affect downstream processes, potentially making conclusions unreliable for many types of cancer analysis. If unbiased VAFs are required, Isaac should be run with soft clipping of semi-aligned reads disabled, or an alternative aligner such as BWA (Li and Durbin, 2009) should be used. Although realignment can be performed where clipped alignments have been previously produced, this may be cost or time-prohibited. For example, projects such as 100KGP have already sequenced and aligned >10 000 tumor-normal genome pairs. In such cases, equally clipping all reads would enable downstream analyses reliant on unbiased VAFs without the need for sequencing data realignment.

While the Isaac aligner version assessed in this study (v03.16.02.19) was released in April 2016, as of November 2019 it is still being used with reference-bias-introducing alt-clipping in 100KGP. Whether reference bias has affected previous studies using the Isaac aligner is difficult to predict. It is clearly essential that aligners, such as Isaac, be evaluated to ensure that the data they produce are not systematically biased.

## Funding

This work was supported by grants from Cancer Research UK [grant number C1298/A8362], Myeloma UK and a David Forbes Nixon Foundation Fellowship (to M.K.).

*Conflict of Interest*: none declared.
